# Lifting and Transport of Martian Dust by the Ingenuity Helicopter Rotor Downwash as Observed by High‐Speed Imaging From the Perseverance Rover

**DOI:** 10.1029/2022JE007605

**Published:** 2022-12-20

**Authors:** M. T. Lemmon, R. D. Lorenz, J. Rabinovitch, C. E. Newman, N. R. Williams, R. Sullivan, M. P. Golombek, J. F. Bell, J. N. Maki, A. Vicente‐Retortillo

**Affiliations:** ^1^ Space Science Institute Boulder CO USA; ^2^ Applied Physics Laboratory Laurel MD USA; ^3^ Stevens Institute of Technology Hoboken NJ USA; ^4^ Aeolis Research Chandler AZ USA; ^5^ Jet Propulsion Laboratory California Institute of Technology Pasadena CA USA; ^6^ Cornell University Ithaca NY USA; ^7^ Arizona State University Tempe AZ USA; ^8^ Centro de Astrobiologia (INTA‐CSIC) Madrid Spain

**Keywords:** Mars, dust, helicopter, Perseverance, Ingenuity

## Abstract

Martian atmospheric dust is a major driver of weather, with feedback between atmospheric dust distribution, circulation changes from radiative heating and cooling driven by this dust, and winds that mobilize surface dust and distribute it in the atmosphere. Wind‐driven mobilization of surface dust is a poorly understood process due to significant uncertainty about minimum wind stress and whether the saltation of sand particles is required. This study utilizes video of six Ingenuity helicopter flights to measure dust lifting during helicopter ascents, traverses, and descents. Dust mobilization persisted on takeoff until the helicopter exceeded 3 m altitude, with dust advecting at 4–6 m/s. During landing, dust mobilization initiated at 2.3–3.6 m altitude. Extensive dust mobilization occurred during traverses at 5.1–5.7 m altitude. Dust mobilization threshold friction velocity of rotor‐induced winds during landing is modeled at 0.4–0.6 m/s (factor of two uncertainty in this estimate), with higher winds required when the helicopter was over undisturbed terrain. Modeling dust mobilization from >5 m cruising altitude indicates mobilization by 0.3 m/s winds, suggesting nonsaltation mechanisms such as mobilization and destruction of dust aggregates. No dependence on background winds was seen for the initiation of dust lifting but one case of takeoff in 7 m/s winds created a track of darkened terrain downwind of the helicopter, which may have been a saltation cluster. When the helicopter was cruising at 5–6 m altitude, recirculation was seen in the dust clouds.

## Key Points

- Six flights of the Ingenuity helicopter were documented with video acquired by Mastcam‐Z on the Perseverance rover in Jezero crater, Mars
- Dust mobilization was expected when the helicopter was below 1‐m altitude but was observed at low wind speeds from >5 m altitude
- Recirculating dust clouds unexpectedly formed while the helicopter was at >5 m cruising altitude

## 1 Introduction

Understanding dust mobilization on Mars is essential for understanding and predicting the background atmospheric dust distribution, the onset and evolution of dust storms (the largest of which can cover the entire planet in a veil of dust for weeks on end, e.g., Guzewich et al., 2019), and the effects of lifted dust on Martian atmospheric dynamics. Due to Mars' low atmospheric density, which absorbs and scatters relatively little solar or thermal radiation, the presence of dust in the atmosphere has a major impact on its radiative balance, which in turn strongly affects thermal gradients and winds (Kahre et al., 2017; Wolff et al., 2017). The global distribution of atmospheric dust depends on atmospheric transport, interactions with ice particles (microphysics), fallout rate, and dust mobilization from the surface. The last process is least understood and in greatest need of investigation (Newman, Bertrand, et al., 2022). Dust lifting has been observed in both straight‐line winds and vortices (dust‐devils), which may be roughly equally important both outside dust storms (Newman, Hueso, et al., 2022) and during the onset of regional storms (Lemmon et al., 2022).

Dust may be mobilized as a direct result of wind stress or indirectly because of other wind‐mobilized particles, and other forces may contribute to lifting. Wind tunnel experiments utilizing flat beds of uniform particles have shown that wind mobilizes fine/very‐fine sand grains (near 100‐μm diameter) more easily than either much finer or coarser particles (e.g., Iversen & White, 1982), discouraging the idea that individual dust‐sized particles might be directly entrained by the wind. Instead, saltation, a bouncing sand grain motion, has been proposed as a prerequisite to dust mobilization, in which easier‐to‐move saltating sand‐sized grains would disturb dust‐sized particles (typically <5‐μm diameter) or aggregates thereof, entraining dust into the turbulent boundary layer (e.g., Greeley, 2002). However, in situ hand lens‐quality images show that surface dust on Mars occurs in resolvable, sand‐sized, but low‐density aggregates with very weak, filamentary structures (Herkenhoff et al., 2004; Sullivan et al., 2008), which should be easier for the wind to mobilize than solid sand grains of equivalent diameter (Merrison et al., 2007). Besides wind stress, Neakrase et al. (2016) identify factors contributing to dust mobilization and entrainment, including pressure back‐venting in the low‐pressure core of vortices (Bila et al., 2020), electrical forces (Kruss et al., 2021), and a thermal‐creep lifting that occurs in atmospheres with long mean‐free paths driven by radiative heating of sediment (Kraemer et al., 2019). In addition, the sand motion may be initialized by turbulent wind gusts and then maintained by lower winds once the motion has begun (Sullivan & Kok, 2017; Swann et al., 2020); this effect is larger on Mars than on Earth (Kok, 2010). Gravity‐dependent cohesive forces within the sand bed could also reduce saltation thresholds on Mars relative to Earth (Musiolik et al., 2018). Low pressures reduce the threshold for saltation by a factor of up to 2.5 (Swann et al., 2020).

The mechanisms that contribute to dust mobilization have not been directly observed on Mars, nor have the threshold wind conditions been directly measured. A direct measurement of friction velocity (related to wind stress by density) was made with Pathfinder's multiheight windsocks, but this experiment did not coincide with any observed lifting or grain motion (Sullivan et al., 2000). Using *Perseverance* data, dust lifting was observed only for instantaneous winds (measured at 1.5 m height) >15 m/s (Newman, Hueso, et al., 2022), while the grain motion occurred within intervals with peak wind speeds (at 1.2 m height) >14.7 m/s in *InSight* data (Baker et al., 2021). Charalambous et al. (2021) used measured ∼1.2‐m height winds and estimates of surface roughness to infer a friction velocity of 2.0 m/s while sand was in motion, but not a threshold. Estimates of threshold wind stress for saltation from sand mobility correspond to 0.7 m/s for the atmospheric densities considered in this work (Ayoub et al., 2014).

Beyond the importance of dust lifting in general on Mars, the specific problem of dust mobilization by rotor downwash is of interest in aviation—in terrestrial helicopter operations, the effect is termed “brownout” (analogous to “whiteout” in snow). In addition to possible degradation of optical sensor performance, lofted dust might cause triboelectric charging (e.g., Farrel et al., 2021; Lorenz, 2020). With helicopters now being considered for sample return operations at Mars, and the development of the Dragonfly rotorcraft for Titan underway, the possibility of extraterrestrial brownout means in situ measurements at Mars have considerable value in testing the generality of brownout models (e.g., Rabinovitch et al., 2021) under physical conditions quite different from Earth.

This paper describes the *Perseverance* rover's video documentation of six *Ingenuity* helicopter flights on Mars over April–September 2021. Each of the flights resulted in dust lifting from the Martian surface at takeoff and landing, and some flights resulted in dust lifting while at 3–5 m cruising altitude. We report on the altitudes for which dust lifting was observed, discuss inferred wind speeds and implications for dust lifting processes on Mars and describe the dynamics of the resulting dust clouds.

## 2 Data and Methods

### 2.1 Flights

**Figure 1:**
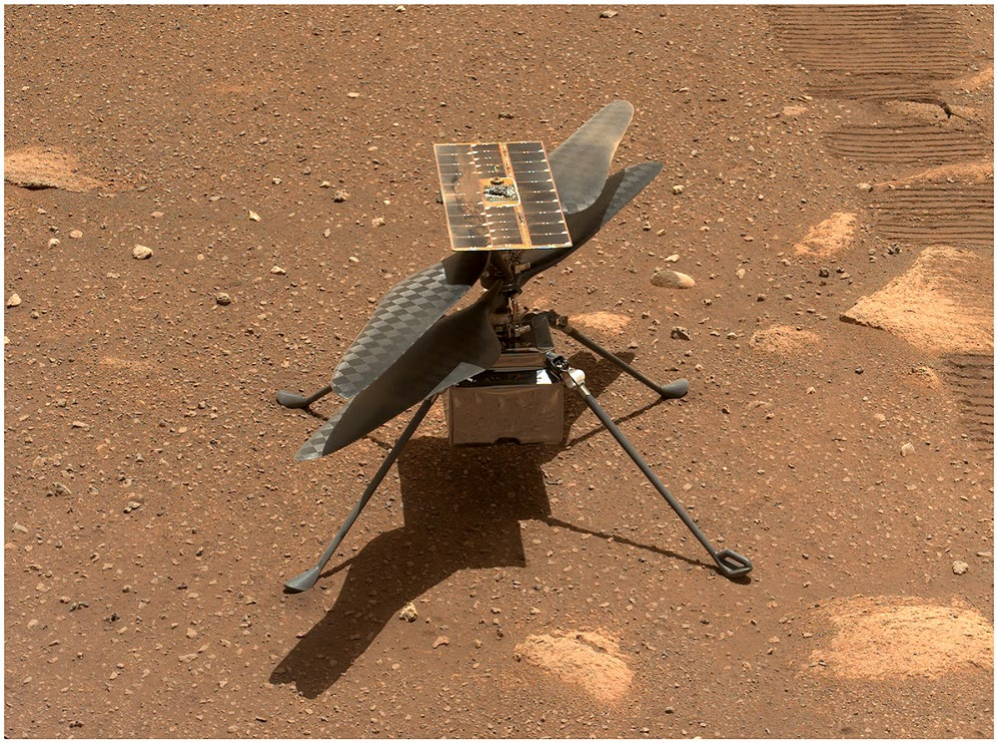
*Ingenuity* on Mars. This sol‐47 Mastcam‐Z image includes the first three landing sites.

*Ingenuity* is a 0.49‐m tall, 1.8 kg rotorcraft that was carried to the surface of Mars by the *Perseverance* rover (Balaram et al., 2021; Lorenz, 2022). It has two counterrotating, 1.21‐m diameter rotor blades, four 0.384‐m legs, a 0.136 × 0.195 × 0.163 m fuselage, and a solar panel (Figure 1). It carries a monochrome camera for navigation purposes and a color “return to Earth” camera for documentation of the Martian terrain. *Ingenuity* was designed as a technology demonstration but was eventually used for reconnaissance in support of the *Perseverance* mission. It was deployed on 3 April 2021, which was Martian day (sol) 44 of the rover mission. Flights were conducted autonomously, using telemetry and navigation camera images to control flight according to presequenced commands. The operation of *Ingenuity* during its first flights is described in detail in Grip et al. (2022).

The first five flights of the *Ingenuity* helicopter on Mars occurred on sols 58, 61, 64, 69, and 76. These flights were the subject of video documentation using Mastcam‐Z for the purpose of obtaining data on flight performance for engineering and documenting the first flights on Mars. After flight 5, a video of most succeeding flights was not taken due to a combination of competing priorities and obstructed view; however, flight 13 was also subject to video recording for the purpose of documenting dust lifting.

**Figure 2:**
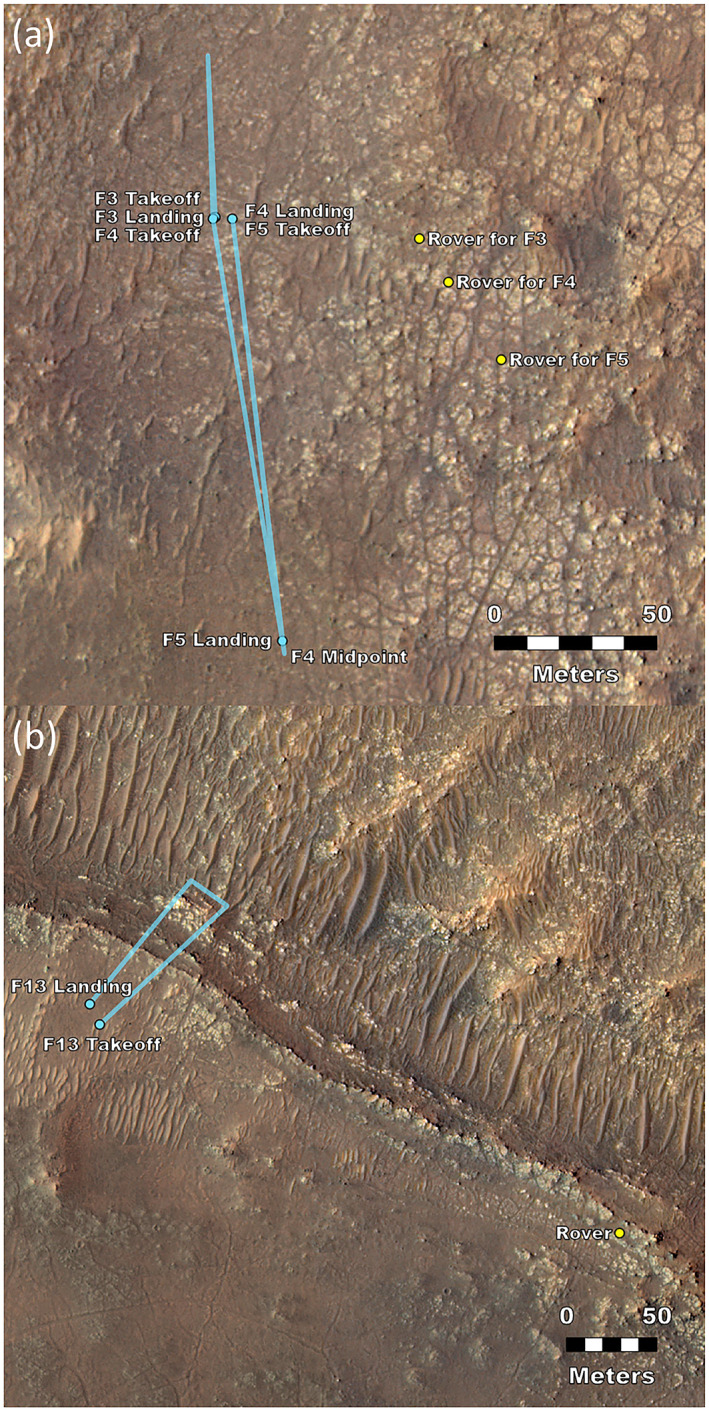
Flight geometry. Locations for flights (a) 3–5 and (b) 13 are shown for the rover, takeoff, and landing sites in orthorectified mosaics (Fergason et al., 2020) of HiRISE images ESP_045994_1985 and ESP_068360 (McEwen et al., 2007). For flights 1 and 2, the rover was at the F3 location, and the takeoff and landing were indistinguishable from the F3 takeoff position shown.

An overview of the flight details is in Table 1. Flight 1 had a rotation and hovered at 3 m above ground level (AGL). Flight 2 had a 2‐m out‐and‐back traverse to the west (away from the rover) at 5 m AGL. Flight 3 had a 50‐m out‐and‐back traverse to the north (out of the video frames) at 5 m AGL. Each of the first three flights took off and landed within a ∼1 m region (Figure 2). Flight 4 was used for reconnaissance of a new landing field, had a 133‐m out‐and‐back traverse 10° east of south at 5 m AGL, and landed several meters from the previous landing spots. Flight 5 was a 130‐m traverse 7° east of south at 5 m AGL, followed by an ascent to 10 m AGL and a landing 110 m from the rover. By flight 13, the rover and helicopter had each moved roughly 900 m to the south‐southeast. Flight 13 was used for reconnaissance to the northeast, with a flight at 8 m AGL and a landing site ∼9.5 m further from the rover than the takeoff site.

**Table 1:** Overview of Flights With Video

Flight	1	2	3	4	5	13
Time (UTC)	19 April	22 April	25 April	30 April	7 May	5 Sept.
(2021)	07:34	09:33	11:32	14:50	19:27	00:08
Sol	58	61	64	69	76	193
Local mean solar time	12:33:13	12:33:20	12:33:21	12:33:22	12:33:17	12:03:57
Duration (s)	38	50	78	117	108	160
Altitude (m)	3	5	5	5	5 (10)	8
Traverse (m)	0	4	100	266	130	210
Distance^a^ (m)	63	63	64 (63)	69 (75)	110	320 (310)
Zoom^b^ (mm)	110, 34	48, 34	26, 26	26, 26	110, 26	26, 110
Rate^b^ ^,^ ^c^ (fps)	6.3, 6.6	6.6, 6.6	6.6, 6.6	6.7, 6.8	15.3, 6.8	6.5, 6.0
P^d^ (Pa)	748	749	746	748	745	698^e^
T^d^ (K)	194	192	194	194	200	239^e^
*ρ* ^d^ (g m^−3^)	20.4	20.7	20.4	20.4	19.7	15.5^e^
W^d^ (m/s)	7.4	9.7	5.3	1.5	3.7	3^e^
Direction^d^	65°	72°	82°	47°	108°	90°^e^

^a^ Distance from rover to landing site (distance to takeoff if observed and different).

^b^ Shown for left and right cameras, respectively.

^c^ Video frames per second.

^d^ Average of Mars Environmental Dynamics Analyzer (MEDA) pressure, 1.45 m‐height temperature, density, and wind data at the rover site over 10 min centered on flight.

^e^ Estimated from nearby sols.

### 2.2 Video

Mastcam‐Z is a multispectral, stereo camera system for documenting the geological and atmospheric conditions around the rover (Bell et al., 2021). Each of the left and right cameras has a zoom lens and could be independently set to 26–110 mm focal length. The resulting resolution is 0.423, 0.212, 0.150, and 0.673 mrad/pixel for the zoom positions of 26, 34, 48, and 110 mm, respectively, that were used for flight video (Hayes et al., 2021). The videos described here were acquired using infrared‐blocking filters that resulted in red, green, and blue images when sampled through the Bayer‐pattern microfilters on the Charge Coupled Device detector (Bell et al., 2021).

**Figure 3:**
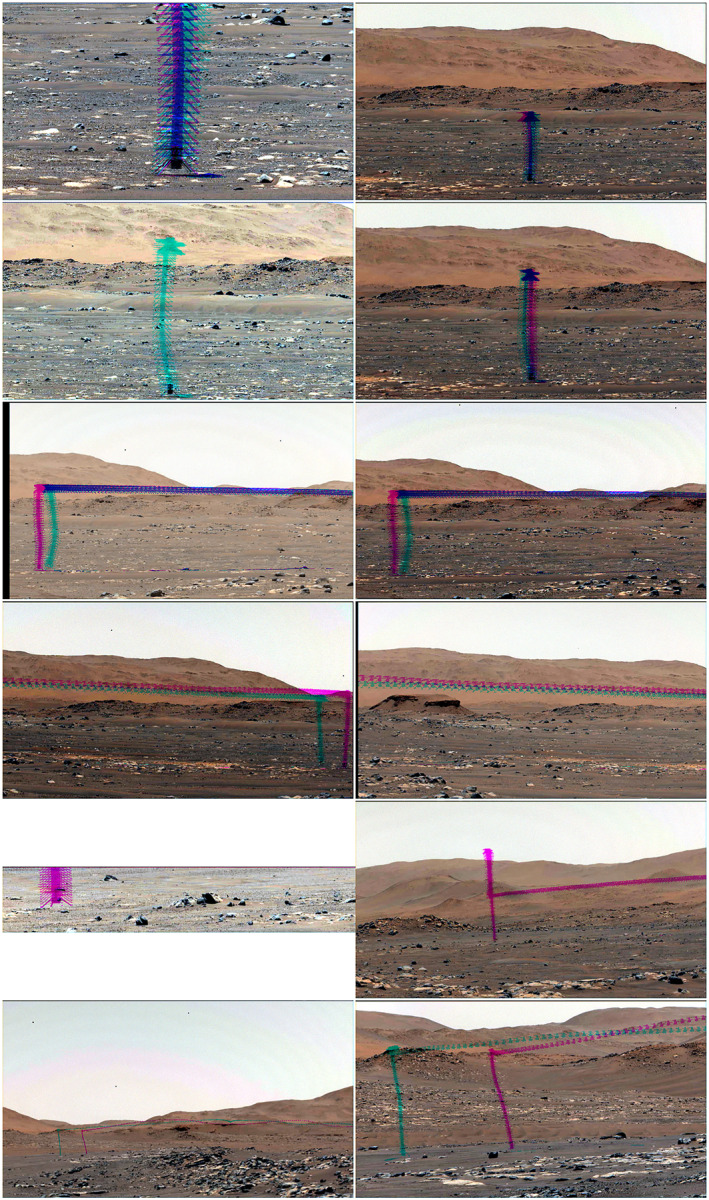
Video summary. Composite images are shown with a 1%–99% color‐stretched mean‐frame image merged with helicopter positions from all frames. Mastcam‐Z left eye (left) and right eye (right) images are shown for flights on sols (from top) 58, 61, 64, 69, 76, and 193. The ascent and the first half of the flight are shown in teal, while the last half of the flight and the descent are shown in magenta; purple results from the superposition.

The flight videos were designed for engineering support and were sequenced to maximize the frame rate within the overall system capabilities. Most videos were taken with 1,280 × 720 pixel subframes of the 1,600 × 1,200 pixel array. They were acquired using real‐time group‐of‐picture compression (Joint Photographic Experts Group [JPEG]), such that each data product was a group of 16 consecutive frames compressed to JPEG quality 50. Most videos were much longer than the flights as a precaution against timing errors. Some video frames were deleted on board without downlink after frames that were of interest were transmitted to Earth. All videos were acquired with both eyes: in some cases, different zoom positions were used to acquire both close‐up images of takeoff or landing and wide‐field images to maximize flight coverage; in some cases, the same zooms were used with offset subframes to increase the flight coverage (Figure 3). For flight 5 on sol 76, the left eye was set to 110‐mm zoom with a 1,600 × 120 subframe for high‐resolution and ‐speed documentation of the landing at a new and distant site. Each pair of videos was acquired with a fixed aim; the remote‐sensing mast was not used to repoint the cameras because tracking the helicopter was not possible.

### 2.3 Additional Imagery and Meteorology

Ingenuity carries two cameras, described by Balaram et al. (2018). One, the Return to Earth Camera (RTE), is a high‐resolution (4,208 × 3,120 pixels), Bayer‐color camera. RTE is a commercial off the shelf (COTS) Sony IMX 214, with a 47° FOV aimed ∼22° below the horizon and was used for occasional images to document the terrain around the helicopter. The nadir‐pointed navigation camera (NAV) is a COTS Omnivision OV7251 with 640 × 480 monochrome pixels and a 133° × 100° FOV.

The Mars Environmental Dynamics Analyzer (MEDA) is a meteorological suite for measuring pressure, temperature, winds, humidity, and radiative fluxes (Rodriguez‐Manfredi et al., 2021). It recorded data from each sensor at 1‐Hz during and around each flight except for sol 193. Table 1 shows 10‐min averages for pressure, 1.45‐m height temperature, and winds; wind azimuth follows the meteorological convention of being the back azimuth, the angle east of north from which the wind comes. Wind speed was computed via vector averaging; it was significantly different from scalar averaging of speed only for the slow winds of sol 69. On sol 193, MEDA was in safe mode and not used; estimates in Table 1 were derived from sols 190 and 194.

Low‐frequency (84 and 168 Hz) sounds of helicopter flights 4, 5, and 6 were detected by a microphone on the SuperCAM instrument (Maurice et al., 2022). No signatures of transport generated at the distance of the helicopter were detected.

### 2.4 Dust Detection

**Figure 4:**
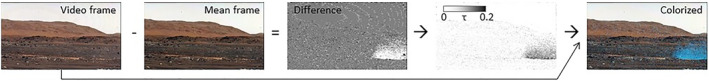
An illustration of opacity derivation. Each video frame was compared to an associated mean frame, resulting in a set of difference images that were converted to optical depth maps. Colorized images enhance the visibility of dust with blue light.

Dust was visible by inspection in raw video images from some flights. To quantify dust lifting and behavior, images were processed to determine the amount of dust and to produce enhanced images for the study of dust motion. The process was similar to dust devil detection and tracking algorithms (Greeley et al., 2006): it required the creation of a mean frame, removal of that mean frame from individual frames, and determination of optical depth from the mean and difference frames (Figure 4). A final step produced colorized images that enhanced the visibility of lifted dust.

For each video, mean frames were initially created from all available video frames; after initial identification of dust lifting, new mean frames were created from dust‐free frames. Such frames were created for times when the helicopter was out of frame when possible and were always created for pretakeoff and postlanding. For each frame, the most appropriate mean frame was used for comparison and differencing.

Optical depth was determined via a low‐opacity approximation of the radiative transfer equation,

(1) $$I=I_{0}\;e^{-\tau }+J_{0}\;*\left(1-e^{-\tau }\right).$$

In Equation 1, *I* is the observed radiance for each pixel and time step, *I*_0_ is the mean‐frame radiance for each pixel, *J*_0_ is the source function convolved with dust scattering properties, and *τ* is the optical depth for each pixel and time step. In principle, *J*_0_ can be determined by analysis of high‐contrast areas of the image with assumptions of locally uniform opacity. In practice, *J*_0_ is nearly equivalent to the nearby sky radiance (Moores et al., 2015), which was used here. Optical depths were derived only for the green channel due to the impact of chroma noise (in the JPEG quality 50 images) on the red and blue channels. Optical depths were typically of the order of hundredths but averaged ∼0.1 for the sol‐64 dust cloud. Colorization was also done via an adaptation of the radiative transfer equation. The mean frame was converted to an enhanced frame by (a) multiplicatively increasing *τ*, and (b) replacing *J*_0_ with a cyan light source, where (a) and (b) were done for each of the color channels.

Estimated dust mass was retrieved from each image by (a) determining the area over which there was an observable dust cloud and no extraneous variability (noise or dust devil); (b) summing optical depth over all pixels in the area; and (c) converting the summed optical depth into dust mass. Because the optical depth is a dimensionless optical cross‐section per unit geometric cross‐section, it includes particle density and the cross‐sectional area and extinction efficiency averaged over the particle size distribution (PSD). For the purposes of the present calculation, a PSD representative of atmospheric dust (mean radius of 1.4 μm and mean variance of 1/3) was used, for which it was determined that the particle volume was 0.8 × 10^−6^ m^3^/m^2^ per unit opacity. For an assumed slightly porous density of 2,000 kg/m^3^, we obtained 1.6 g/m^2^ per unit opacity. Intact, pseudo‐spherical aggregates (e.g., 250‐μm radius and 380 kg/m^3^ density) would result in an order of magnitude larger estimate for mass; however, the expectation of disaggregation during entrainment (whether by a violent saltation event or due to wind shear directly destroying the aggregates) led us to choose suspended atmospheric dust, rather than aggregates as the appropriate analog for lifted dust.

## 3 Results

### 3.1 Dust Amount

**Figure 5:**
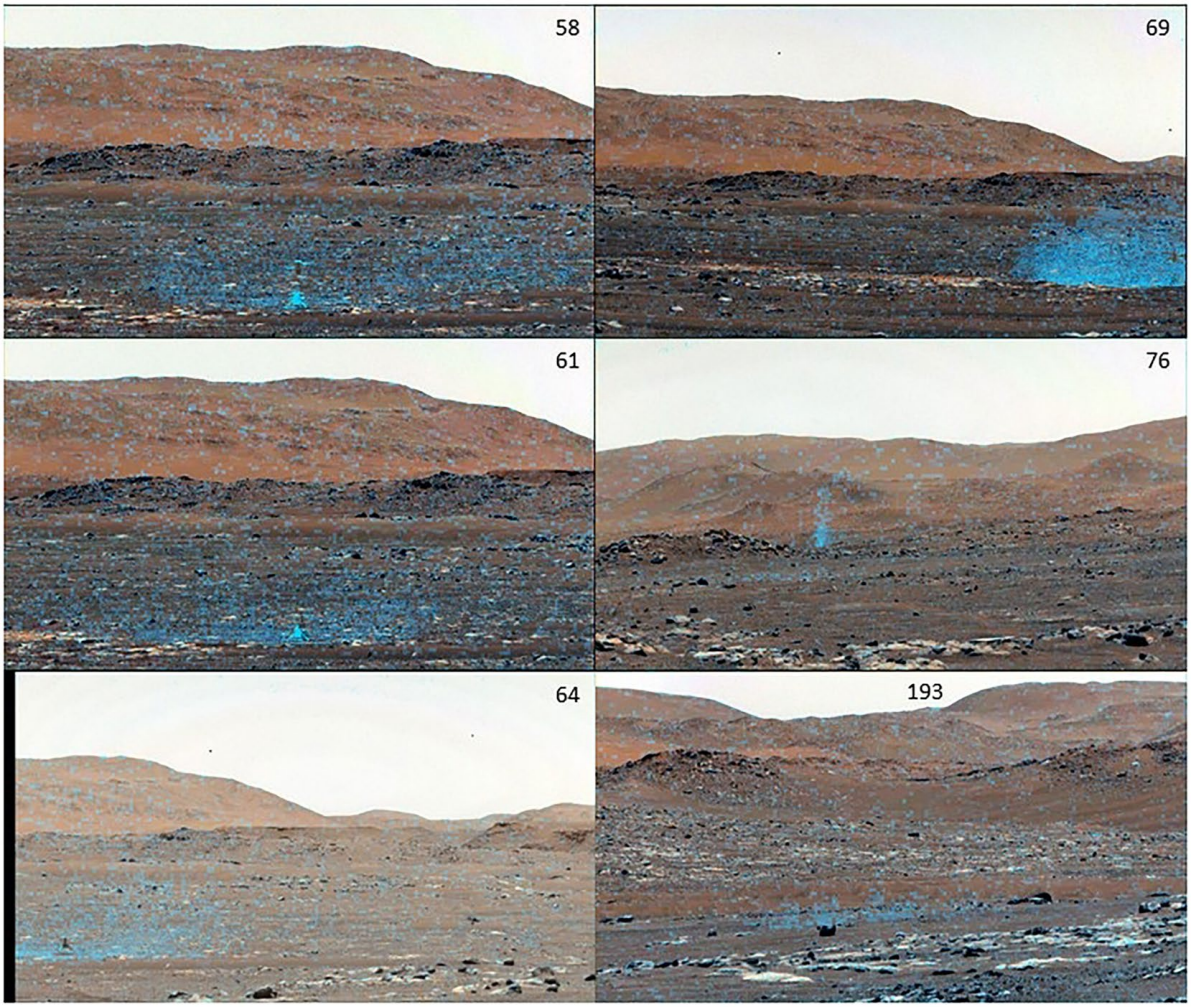
Dust clouds at and near landing, with colorization. Sol number of flights is indicated for each image.

**Figure 6:**
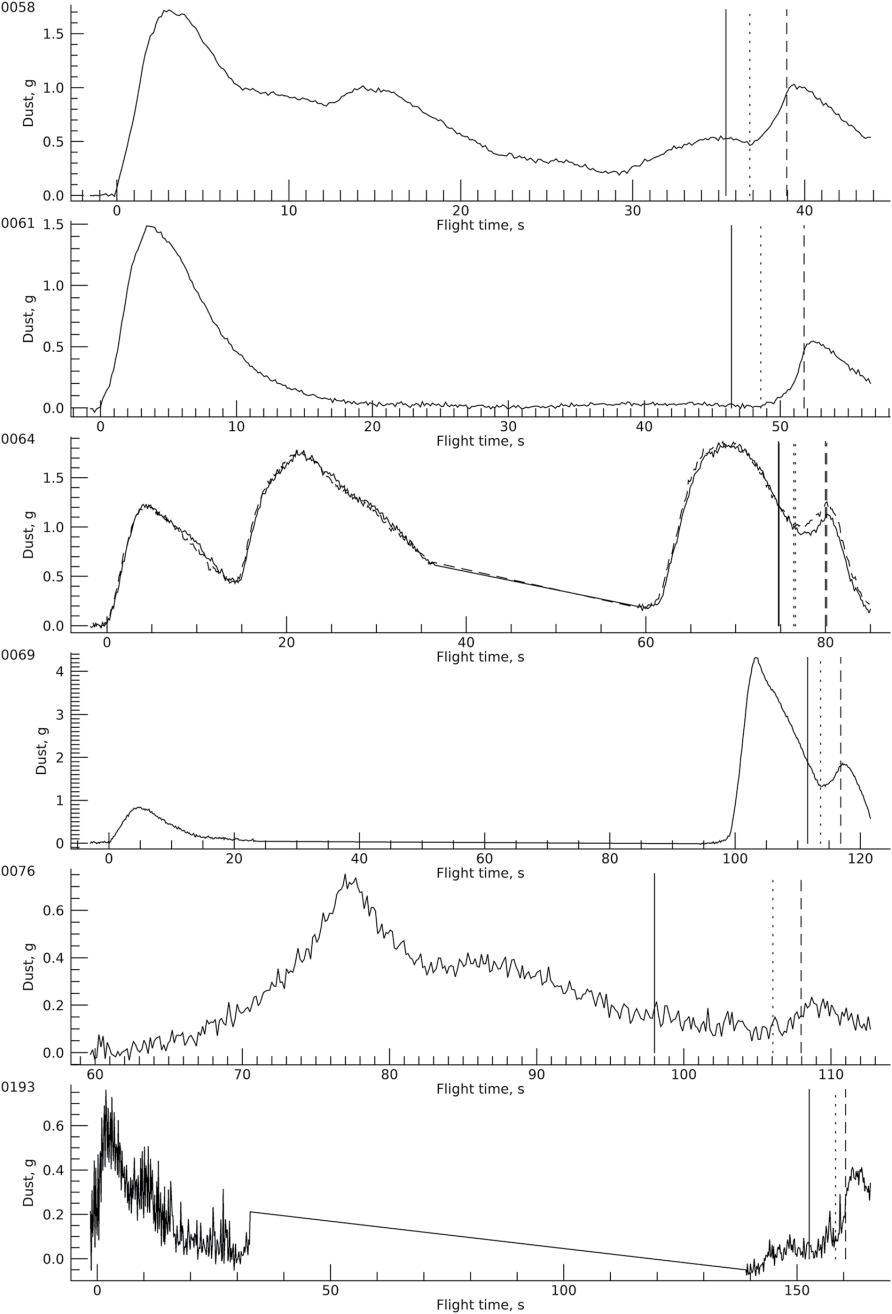
Suspended dust mass over time. Vertical lines mark the start of constant‐speed descent (solid), visually detected dust lifting under the helicopter (dotted), and touchdown (dashed). The curves for sols 58, 61, 76, and 193 were obtained with the right eye, sol 69 with the left eye, and sol 64 with each eye (right eye dashed). From sol 64, there were times with the helicopter out of the field of view that were not measured.

All movies that showed sufficient area around the lander and included the sky were processed to determine opacity. Figure 5 shows examples of the resulting enhanced images near each observed landing. Note that the sol‐76 example includes a dust devil in the background. The summed opacity, pixel FOV, and distance were used to determine the total suspended mass over time (Figure 6). The absolute value of the suspended mass is subject to factors of several uncertainty due to the specific choice of atmospheric dust parameters; relative changes are more accurate as long as the dust stays in the frame and at the same distance. In practice, the dust does neither, and the suspended dust measurement always declined after initial lifting due to a combination of motion out of the frame and increasing distance (since projected pixel area scales with the square of distance).

Takeoff is affected by bringing the rotors with the blade set at low incidence angles (low thrust) up to operating speed (2,537 rpm) and then increasing the collective pitch to near‐instantaneously increase the thrust. The thrust:weight (T:W) ratio initially exceeds one to leave the ground, then the helicopter ascends at ∼1 m/s. For example, data from flight 1 (Lorenz, 2022, Figure 6.9) show the collective angle, and thus thrust, had a brief (<1 s) pulse about 30% higher than hover values in order to break cleanly away from the surface, and then a couple of seconds at a thrust ∼20% higher than hover in order to perform the commanded ascent (see also Grip et al., 2022). Generally, lifted dust was seen on the last frame with the helicopter on the surface, and dust lifting continued for ∼3 s. All flights except 2 and 13 showed significant dust lifting while at the hover/transverse altitude. Descent involved rapid downward acceleration followed by a constant velocity (T:W ∼1) descent to touchdown.

### 3.2 Flight Narrative

#### 3.2.1 Sol 58

Flight 1 caused the most observed dust lifting on takeoff, ∼1.7 g. About 12 s into the flight, the helicopter turned in place and adjusted the thrust vector to respond to the wind and maintain the station. This caused dust lifting adjacent to the area immediately below the vehicle. At 29 s, the helicopter responded to a wind, causing a new burst of off‐center dust lifting. Initial winds from the east‐southeast carried the takeoff dust cloud to the right (rover‐measured winds were from ENE). At landing, easterly winds carried dust away and to the right. Notably, the preflight and postflight mean‐frame images differed in that a darkened streak appeared along the trajectory of the takeoff dust cloud; dust removal continued downwind of the helicopter and formed a dust‐cleaned track (see Supporting Information S1).

#### 3.2.2 Sol 61

Flight 2 had a large burst of dust lifting on takeoff and less dust lifting on landing at the same location. There was no observed dust lifting during the hover or the ∼2 m out‐and‐back traverse. Based on the dust cloud motion, winds were away (E) and to the left (S), consistent with rover winds from the ENE. No track was observed. Incidentally, a vortex was detected at the rover 63 m away, 5 min before takeoff.

#### 3.2.3 Sol 64

Flight 3 had comparable dust lifting on takeoff and landing as seen by both eyes. The small difference in the observed landing profiles (Figure 6) was due to the dust moving out of frame sooner for the left eye; otherwise, the profiles were encouragingly similar. The novel aspects of flight 3 were the first traverse out of frame, and the first dust lifting along a traverse. A dust removal track was observed under the flight path. Winds measured at the rover were from the east, while the observed motion was east and north on takeoff and east and south on landing. A vortex passed the rover while moving toward the helicopter ∼20 s postflight, with an observed 1 Pa pressure drop, 7–13 m/s counterclockwise winds, and no obvious sign of dust (c.f. Newman, Hueso, et al., 2022).

#### 3.2.4 Sol 69

Flight 4 had no observed dust cloud along the outbound traverse. Two rover‐Navcam images taken while the helicopter was out of the Mastcam‐Z field of view also showed no dust lifting. When the helicopter reappeared in the frame, it was within a recirculating dust cloud that moved with the helicopter at ∼3 m/s (to the right within the frame). During landing, the helicopter continued to circulate dust from this cloud. After landing, the dust cloud departed at ∼1 m/s to the north, although the takeoff dust cloud went west, and a distant dust devil moved north; rover‐measured winds were slow and varied, mostly from the northeast. A dust removal track was observed under the flight path.

Note that while the average wind over 10 min around Flight #4 was quite low, there were strong turbulent gusts reaching ∼10 m/s at the beginning of the flight. These gusts caused appreciable low‐frequency acoustic noise (see Extended Data Figure 1b of Maurice et al., 2022).

#### 3.2.5 Sol 76

Takeoff was not observed. After the helicopter came in the frame, dust was lifted along the traverse. The cloud largely dissipated when the helicopter ascended to 10 m, and a smaller dust cloud was raised on landing. Rover‐measured winds were from the ESE, several distant dust devils moved to the right (NW to W), and the dust clouds lifted by the helicopter departed away and left (to S).

#### 3.2.6 Sol 193

Dust clouds were raised only on takeoff and landing. The dust clouds moved away and slightly to the right, consistent with easterly winds at the rover.

## 4 Discussion

### 4.1 Dust Lifting During Landing

**Figure 7:**
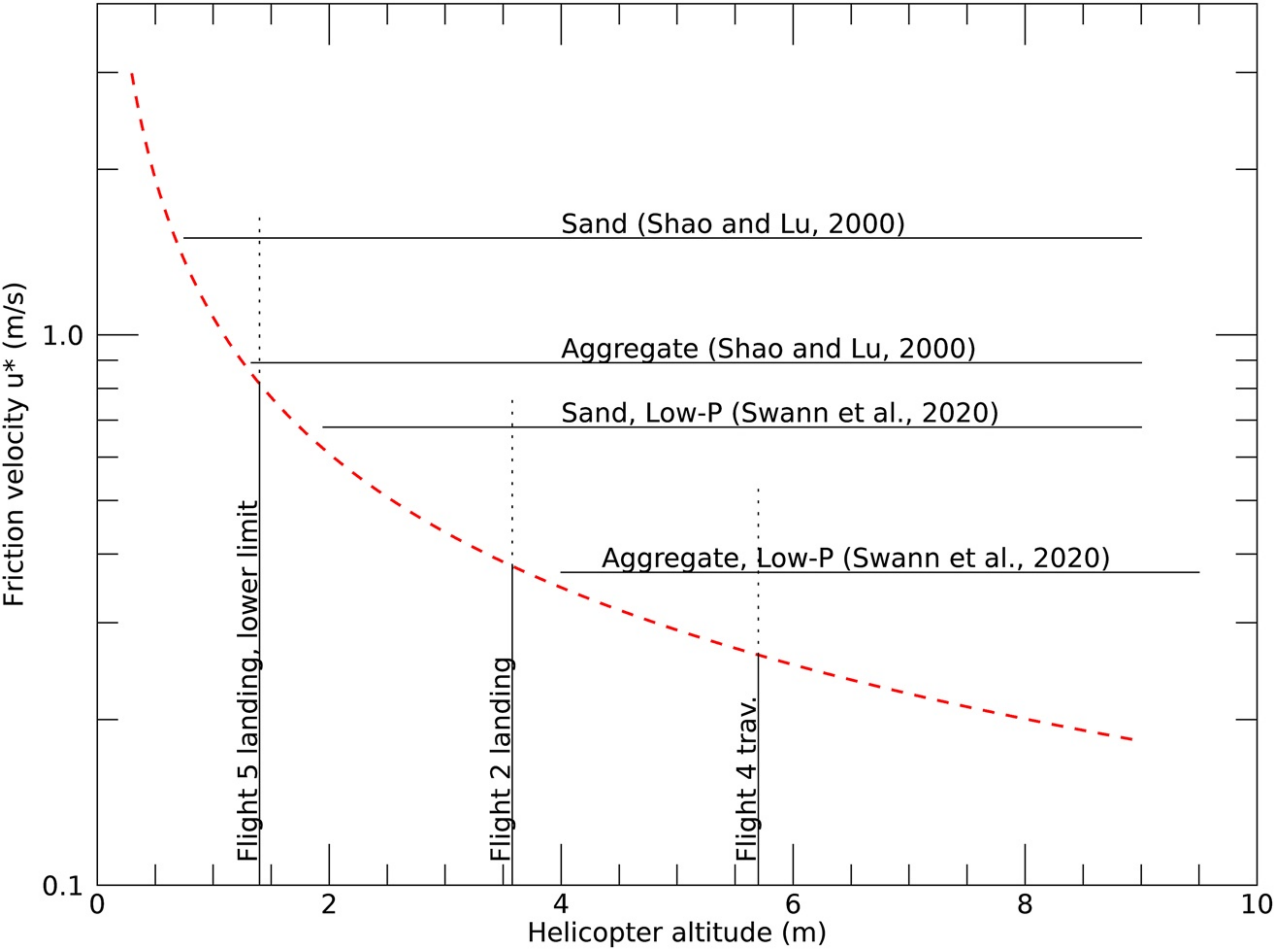
Friction velocity and helicopter altitude. The Rabinovitch et al. (2021) model, adapted for atmospheric density of 0.020 kg/m^3^, 2,800 rpm, and Thrust:Weight = 1, is shown as a red dashed line. Horizontal lines show representative thresholds for a conventional model (Shao & Lu, 2000) and a low‐pressure model (Swann et al., 2020); the calculated thresholds are for mobilization of sand (200 diameter, 3,200) and aggregates (500 μm, 380 kg/m^3^). Vertical solid lines show representative altitudes at which dust lifting was seen during landing and traverse; dotted lines are extended upward to 2x the model prediction.

To assess the winds associated with the observed dust lifting, we relied on the helicopter brownout model of Rabinovitch et al. (2021), which was developed early in the Ingenuity development cycle to estimate the expected severity of sediment mobilization during landing with a constant descent velocity. The brownout model of Rabinovitch et al. (2021) uses several simplifying assumptions that allow the friction velocity on the Martian surface (generated by the high‐speed helicopter rotor wash flow interacting with the surface) to be analytically predicted as a function of the helicopter altitude, helicopter operating parameters, and Martian environmental conditions. The brownout model includes the bulk rotor‐driven wind‐field and the advection of tip‐vortices shed by the rotors. While a computational fluid dynamics (CFD) model would allow a fuller investigation that was out of the scope of this effort. Figure 7 shows the predicted friction velocity immediately under the helicopter (at one rotor radius) using the Rabinovitch et al. (2021) analytical framework with input parameters of atmospheric density = 0.020 kg/m^3^, spin rate = 2,800 rpm, and T:W ∼1 conditions. We did not model takeoff due to expected large departures from equilibrium, high T:W, and the difficulty in determining when dust lifting ceased. Furthermore, the initiation of dust lifting was easier to identify during landing compared to termination of dust lifting during takeoff. To gain confidence in the analytical framework, model predictions were compared to experiments with an analog helicopter on Earth (see Supporting Information S1) and showed the predictions to be accurate to within a factor of 2 with the model overpredicting wind speed.

To determine the friction velocity at which dust lifting initiated, we initially inspected the images that first showed lifted dust and measured the altitude from the midpoint of the rotors to the surface at touchdown (in flight this was ∼30 cm larger than the commanded cruise altitude). To refine our visual estimate of the altitude at which dust lifting initiated, and determine uncertainty therein, we estimated the lifted dust over time. For this, we assumed dust lifting to be 0 when the Rabinovitch et al. (2021) predicted u* to be below the threshold, and otherwise proportional to (u* − u*_t_)·u*^2^, where u* was the altitude‐dependent prediction and u*_t_ was the desired threshold friction velocity (e.g., Kawamura, 1951). The new calculation also allowed for loss over time, parameterized as a single decay rate but representing dust that drifted out of the frame, that drifted away, and that sedimented to the surface. The results included a fit u*_t_ that was dependent on the Rabinovitch et al. (2021) predictions; robust altitude determination (i.e., one that would have resulted from any reasonable model of friction velocity vs. altitude); and a dust loss rate for which we expect geometric effects to dominate the sedimentation term.

**Figure 8:**
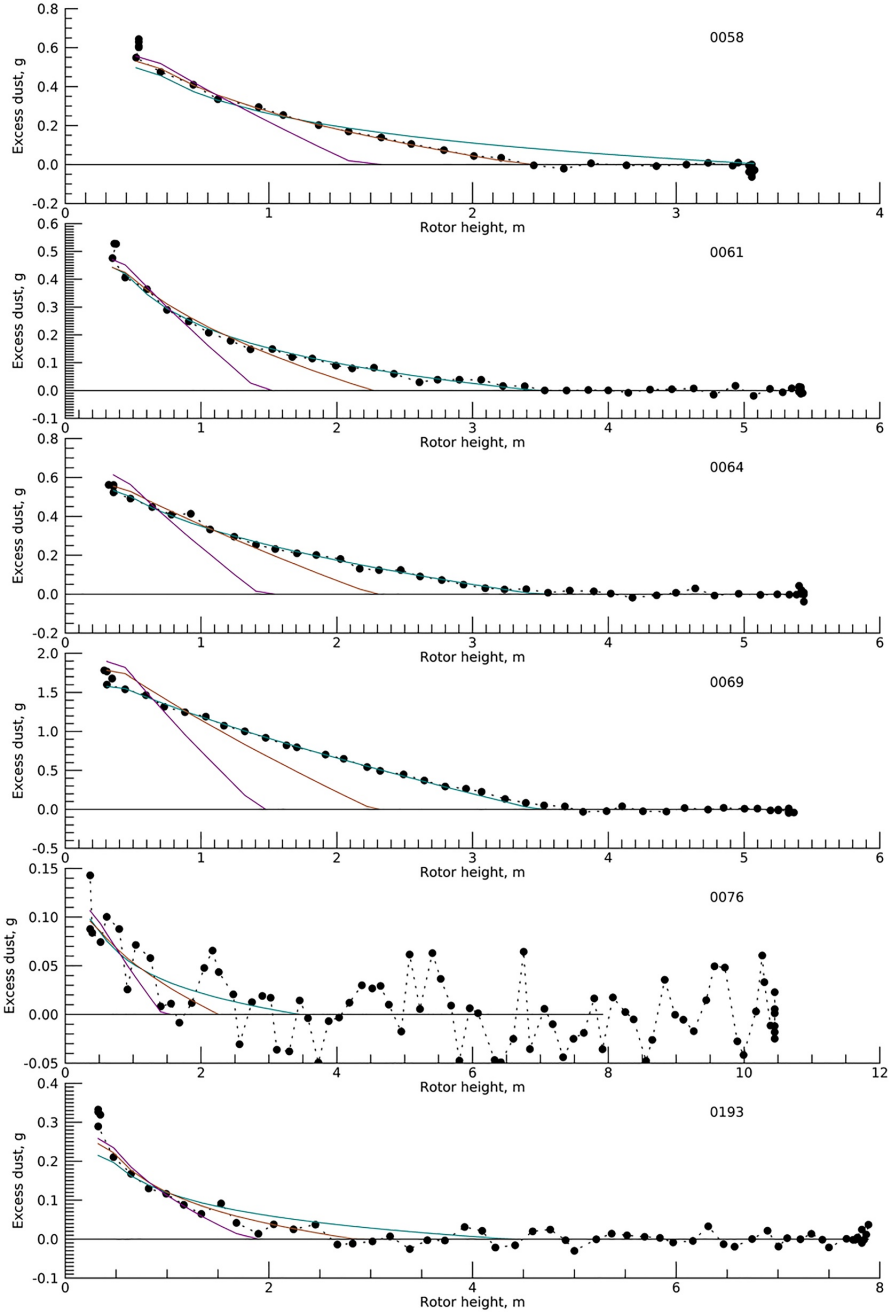
Lifted dust mass versus altitude for landing dust cloud after removing background dust. Colored curves are best‐fit models for u*_t_ = 0.8 (purple), 0.55 (brown), and 0.39 (teal) m/s.

Figure 8 shows the dust within the landing area as the helicopter descended for all six flights, selected based on pixels that contained the dust cloud at some point near the landing. Clusters of points at high altitude represent times immediately before the descent, while those at low altitude show the changing dust at near‐constant altitude (the helicopter tended to bounce slightly as it contacted the surface at 1 m/s). The threshold velocity was fit for each case; for illustration purposes, Figure 8 shows fits for u*_t_ = 0.8, 0.55, and 0.39 m/s. Table 2 shows each individual solution along with the corresponding altitude.

**Table 2:** Dust Mobilization Observations

Flight	1	2	3	4	5	13
Takeoff						
Plume radius (m), t/o+2s	6.1	6.1	5.4	>7.4	n/a	>4.6
Plume height t/o+2s	2.1	2.1	2.1	2.4	n/a	1.8
M_dust_ (g), t/o+2s	1.65	1.19	0.60 (L), 0.74 (R)	0.50	n/a	0.4
U (m/s)	4.1	4.1	5.3	6.0	n/a	4.6
Landing						
Plume radius (m)	4.7	5.7	4.2	6.3	n/a	3.9
Plume height (m)	2.6	2.5	3.4	3.2	n/a	0.7
M_dust_ (g)	1.13	0.81	0.70, 0.59	1.99	0.3	0.42
Z_lift_ (m)^a^	2.32 ± 0.24	3.58 ± 0.24	3.47 ± 0.22 (L) 3.06 ± 0.32 (R)	3.6 ± 0.1	>1.4	2.8^−0.7^/_+1.2_
u*_t_ (m/s)^a^	0.54 ± 0.03	0.38 ± 0.02	0.39 ± 0.02 (L) 0.43 ± 0.04 (R)	0.38 ± 0.01	<0.8	0.56 ± 0.14
Shear stress *τ* (Pa)	0.006	0.003	0.003 (L) 0.003 (R)	0.003	<0.013	0.005

^a^ Uncertainties refer only to the model parameter estimation and do not include the generally more significant factor of ∼2 from model validation.

The modeled winds and observed altitudes require some dust lifting by mobilization and destruction of aggregates. Dust lifting on landing always occurred at altitudes comparable to or higher than the highest altitude for which sand saltation would be expected, as shown by the comparison of model winds and the low‐pressure sand saltation model of Swann et al. (2020) in Figure 7. The first four landings show dust lifting at friction velocities below the threshold for sand mobilization but near or above that for low‐density aggregate analogs (Merrison et al., 2007).

Analysis of dust within an even smaller region immediately around the landing site produced similar results for all but flight 4 (see Supporting Information S1). For flight 4, the initiation of dust lifting under the helicopter was not visually distinguishable from the recirculating cloud, and the fit seemed influenced by spatial variations within the cloud that existed before descent. Thus, while the inflection in total dust amount over time was consistent with data from sols 61 and 64, the fit was judged less reliable.

A notable feature of the dust lifting shown in Figure 8 is that it did not turn sharply up as the helicopter approached the touchdown. In the Rabinovitch et al. (2021) model, u* scales with ∼*h* ^−0.8^ where *h* is the rotor height, and the drag force on particles scales as u*^2^ (Greeley & Iversen, 1985).

### 4.2 Dust Lifting During Takeoff

**Figure 9:**
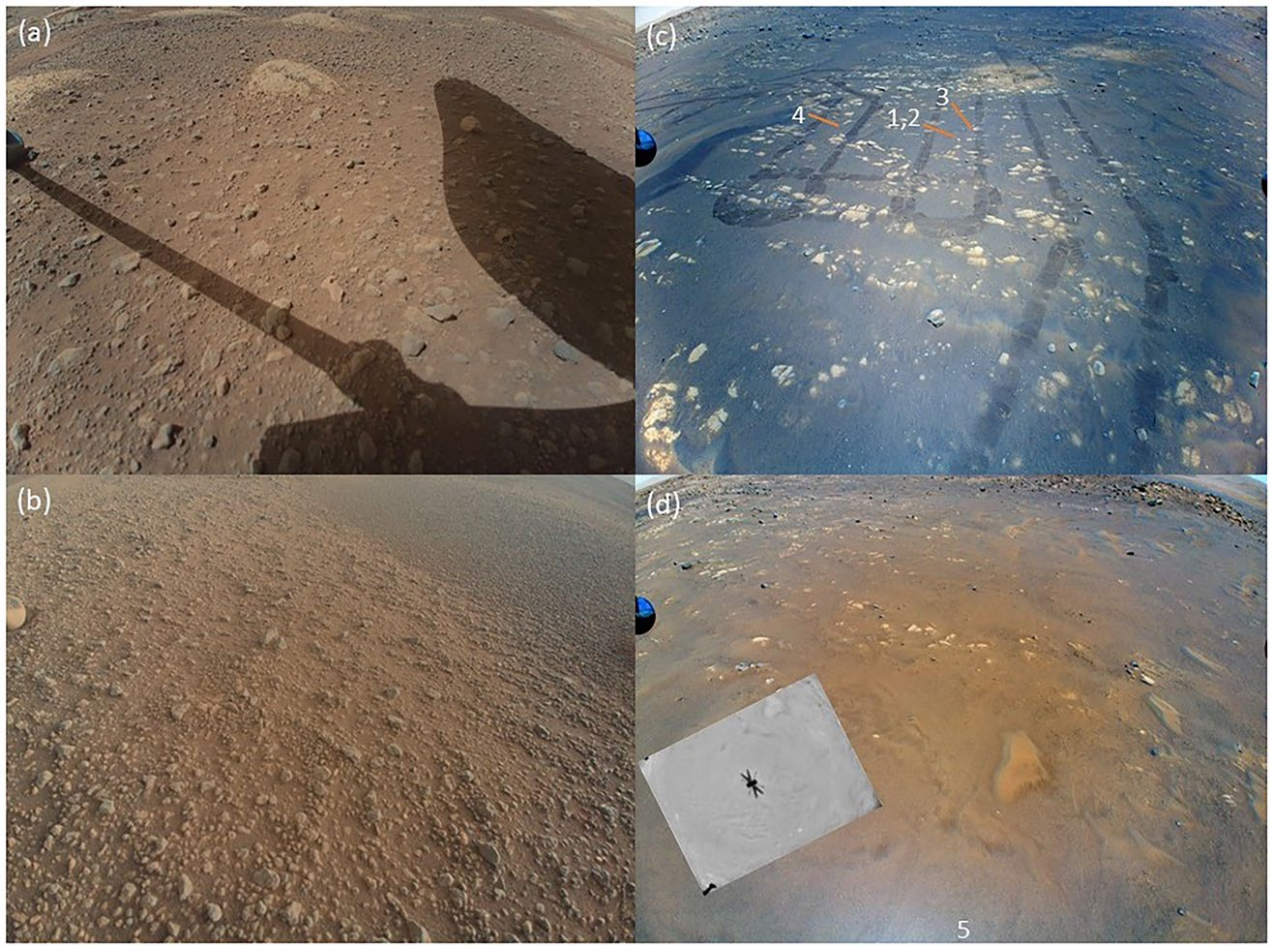
Landing site characteristics in Return to Earth Camera images: (a) sol‐46 image prior to flight 1; (b) sol‐214 image from flight‐13 landing site; (c) sol 64 image from the return traverse, showing landing sites 1–4 and disturbed terrain; (d) sol‐76 image with nadir‐pointed navigation camera inset showing flight‐5 landing site.

Table 2 also reports the size of the dust clouds 2 s after liftoff and at touchdown and a measurement of dust motion. In the first frame with visible lifted dust, the diameter of the dust outline along the surface was measured. The size was measured in succeeding time steps. The rate of expansion of the dust lifting front in Table 2 corresponds to the rate for the first two time steps; the expansion of the dust cloud slowed from frame to frame. The initial expansion was 4–6 m/s within the bottom ∼5 cm of the atmosphere. We note that dust lifting in winds of those speeds is consistent with the landing measurements for surface roughness values of order 1 mm; however, they are only consistent with the absence of dust lifting for 1.5‐m winds <15 m/s (Newman, Hueso, et al., 2022) for surface roughness of 1 cm or greater. The actual operations areas (Figure 9) show terrain consistent topography that is rough at mm‐scale (e.g., coarse sand), cm‐scale (pebbles), and larger scale (rocks) in varying mixtures. For dust devils, sparse obstacles can result in an optimal roughness that results in high sediment flux compared to sand alone or to high density of cm‐scale roughness (Neakrase & Greeley, 2010). We note that flights in such terrain may implicitly result from choosing airfields with a low density of obstacles in an environment where zero‐density of obstacles is not available. The Rabinovitch et al. (2021) model does not include the effects of varying the density of different roughness scales, which may be of interest in future experimental CFD work.

Track formation from the removal of bright dust results in surface darkening (Charalambous et al., 2021). This was seen under the helicopter's path for two traverses. Within recirculating dust clouds traveling with the helicopter, dust lifting rates were difficult to measure during the traverses. Dust removal may have been as high as 0.01–0.1 g/m^2^, and the resulting contrast (in a heavily foreshortened view) was 2%–3% darker for flight 3% and 3%–4% darker for flight 4, similar to typical 2.5% drops for dust devil tracks (Reiss et al., 2016). Since a monolayer of 3‐μm dust would have a mass that is orders of magnitude less, either the dust removal was lower (e.g., spread over a larger area) or the contrast was low due to substantial remaining dust. Notably, one track, on flight 1, did not correlate with the helicopter's position and instead moved downwind from the ascent/descent site. This may have been a saltation cluster, indicating that saltation occurred at least during takeoff when u* may have been ∼2 m/s and then continued downwind, within lower wind speeds.

### 4.3 Dust Lifting During Traverses

Dust lifting was seen on 4 of 5 traverses (among those that exceeded 2 m length) at cruising altitude of 5 m AGL (5.1–5.7 m rotor height). One traverse at 5 m and both at 8 m had no observed lifting. For flight 3 outbound and inbound and flights 4 and 5 inbound, the model‐predicted u* was 0.26–0.29 m/s.

While the landing sites (Figure 9) were selected based on smooth surfaces and other enabling characteristics, the traverses included more diversity of terrain. We note that much terrain that was overflown did not produce dust lifting; heterogeneity of characteristics such as dust availability and roughness resulted in heterogeneity of lifting outcomes. The flight‐3 lifting was seen while the helicopter was over tracks that the rover had made (see bottom of Figure 9c), while the other events were over terrain that was undisturbed or had been flown over from 5‐m AGL.

Dust lifting on traverses requires mobilization and destruction of aggregates, as sand mobilization thresholds are not approached even if the model underpredicts by a factor of 2. Lifting occurs above the expected altitude for mobilization of aggregates (Figure 7). A possible explanation is that some aggregates are not compact spheres, as tested by Merrison et al. (2007), and filamentary aggregates may be more easily destroyed when they intrude into winds.

### 4.4 Implications for Threshold Velocity and Dust Lifting Mechanisms

Although there was only a small sample of observed dust lifting conditions, several observations are important. First, flights 5 and 13 were the only landings in undisturbed terrain. They had u* ∼0.6–0.8 m/s, or wind stress *τ* ∼5–13 mPa. Second, flight 1 exhausted its dust source from 3.3‐m height  but found a new source at ∼6 mPa during descent. Third, flights 1–3 all landed in disturbed terrain—not just the rover tracks visible in Figure 9 but also the effects of taking off from the same site. They had u* ∼0.4–0.5 m/s or surface shear stress ∼3–6 mPa. Fourth, some areas had dust lifting at u* ∼0.26–0.29 m/s or wind stress ∼1.4–1.7 mPa.

These and earlier observations constrain dust lifting mechanisms in several ways. First, multiple mechanisms (or a significant variation in conditions) must have been relevant. Dust lifting occurred with 4x less wind stress during parts of traverses than at any landing site. Dust lifting stalled and then restarted during the first landing. Second, saltation likely played some role, at least in high background winds. Specifically, the flight‐1 takeoff appeared to initiate a saltation cluster that propagated downwind. Third, dust lifting was easier (had a lower threshold) in disturbed material (such as rover tracks or earlier flight paths) than in undisturbed material. This is unsurprising and consistent with observations of more sediment motion in disturbed versus undisturbed material in a dust storm (Lemmon et al., 2022). Fourth, sand mobilization cannot explain all dust lifting. The predicted friction velocity for dust lifting on landing in undisturbed terrain was similar to predictions for sand mobilization at low pressure; and for undisturbed terrain, the predicted friction velocity was within a factor of two of mobilization thresholds. Along traverses, dust lifting occurred at <0.3 m/s and cannot be explained with sand mobilization. It is likely that some places along the traverse had a high dust content, and in those cases the dust formed a large mix of compact (Merrison et al., 2007) and filamentary (Sullivan et al., 2008) aggregates. This may reconcile observations of extensive lifted dust in dust devils and gusts with low abundance of sediment mobilizing events at the rover (Newman, Hueso, et al., 2022).

Note that wind flow influences the dust mobilization and distribution in ways we did not model. Greeley et al. (2003) showed that the vortical motion within dust devils enhanced dust lifting compared to straight‐line winds. The brownout model includes tip vortices but only via the increased wind speed; pressure effects may contribute to enhanced lifting. Further, the brownout model does not include the vorticity effects of counterrotating blades. Future CFD simulations which can resolve the complex rotor‐wake/ground interactions may allow more specific sediment threshold mobilization constraints to be determined from these observations.

### 4.5 Dynamical Behavior of Lifted Dust

**Figure 10:**
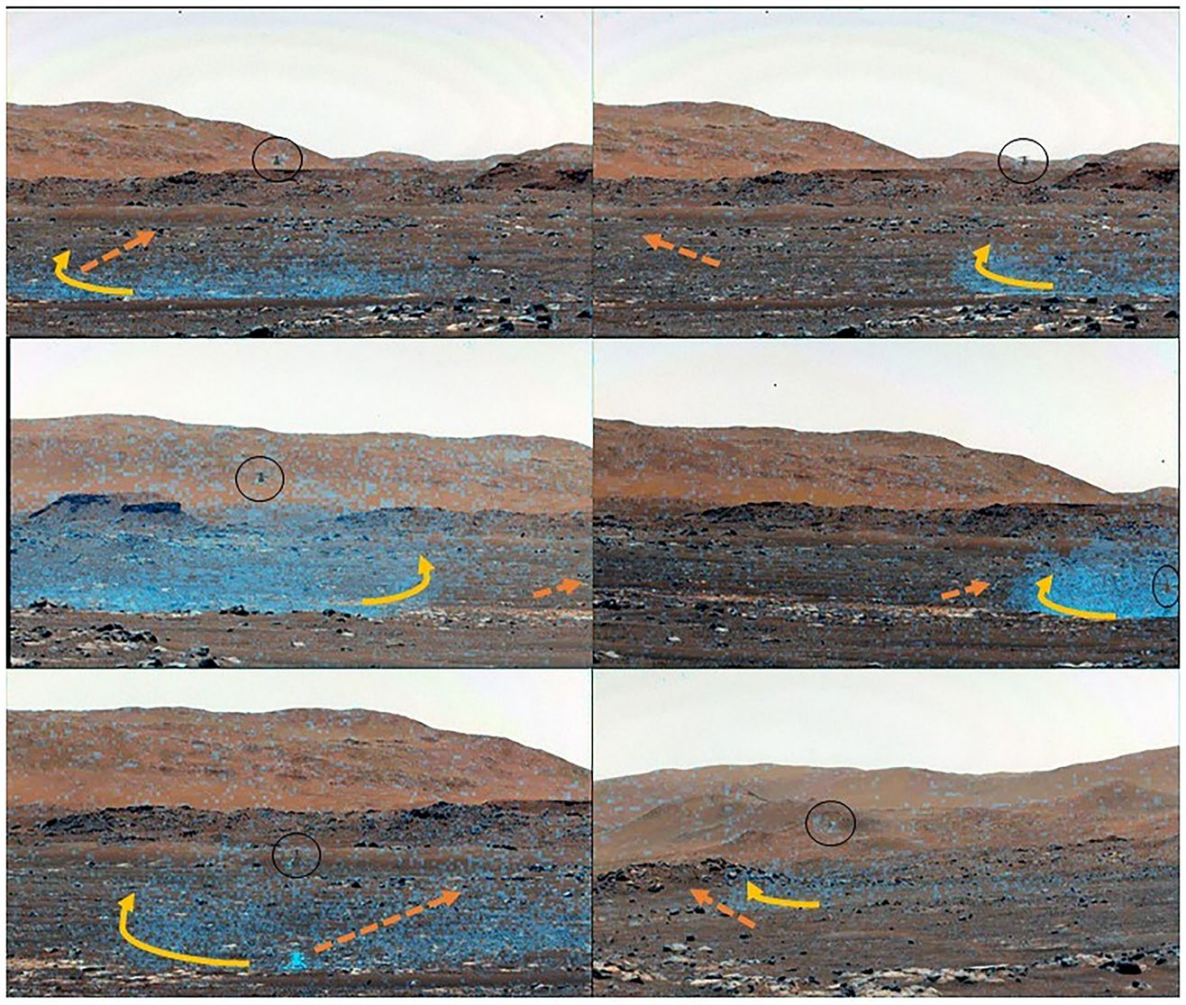
Dust clouds during the traverse. Top: sol‐64 outbound flight and return flight (from left); Middle: sol‐69 traverse and landing; Bottom: sol‐58 hover and sol‐76 traverse. Orange dashed arrows represent winds as seen from dust cloud advection. Yellow solid curves represent internal dust cloud motion. Circles show Ingenuity position.

When dust clouds formed, they expanded initially in all directions regardless of wind direction and speed. Within 2–3 s, the clouds elongated downwind with diffuse boundaries, and a curved and distinct boundary (front) was visible on the windward side. Pulses of lifted dust at takeoff and landing drifted away and became diffuse within seconds. Figure 10 shows examples of dust cloud motions. (See also Movies S1, S2, S3, S4, S5, S6, S7, S8, S9, S10, S11, and S12).

For terrestrial helicopters (Lorenz, 2020) and our observations, a low‐altitude, nadir‐directed wind flow is radially redirected outward near the surface, turns upward as that flow is resisted by the environment, and returns to the rotor intake area to form a recirculation pattern. For the flight‐4 dust cloud, there is a distinct recirculation pattern as dusty air was circulated up to the rotor height and pushed back down. The flight‐3 dust clouds also had the beginnings of such recirculation as tendrils of dust reached above the helicopter. In the flight‐4 case, the recirculation organized the dust cloud around the helicopter: during the traverse, the cloud moved at the speed of the helicopter; once the helicopter had landed, the cloud drifted away at 1/3 of the previous speed. During approach and landing, new dust was lifted, and the recirculating pattern narrowed as the rotors descended.

The Lorenz (2020) flux balance model, while it shows reasonable agreement with a set of dust loading measurements for several different helicopters, and with a set of dust density measurements for a hovering helicopter at different altitudes (Rodgers, 1968), predicts dust concentrations for *Ingenuity* about an order smaller than what we observe here. The model in principle incorporates planetary environmental factors, in that the dust emission flux is scaled by atmospheric density times a flow velocity cubed, and that the sedimentation velocity of the suspended dust takes gravity, particle and fluid densities, and particle size into account. Here, we use the sedimentation velocity observed via the cloud decay. Yet while the basic physical framework of the model seems applicable, the parameterization of dust flux with downwash velocity (derived from field measurements on Earth) requires adjustment for Mars application.

It is interesting to observe that the dust cloud masses we determine are of the order of 2 g, or about 1/1000 of the mass of the Ingenuity vehicle itself. This may be compared with the dust cloud masses estimated for terrestrial helicopters in the “Sandblaster 2” field tests using the data summarized in Table 2 of Lorenz (2020). For the largest helicopter consider, the CH‐53, the dust cloud (approximated as disk area times rotor height times dust density) approaches some 5 kg. All five cases for which data were available (UH‐1, CH‐46, HH‐60, CH‐53, and V‐22) had dust clouds of 0.07–0.2 thousandths of the vehicle mass, indicating that while the dust clouds we have observed on Mars are small and thin, their generation is disproportionate given the tiny weight of *Ingenuity*.

## 5 Conclusions

We observed dust lifting during the takeoff, traverse, and landing phases of six helicopter flights. During landings, the onset of dust lifting occurred when the helicopter was at altitudes of 1.4–3.6 m, with higher altitudes associated with more disturbed terrain. During 4 out of 5 traverse legs at 5–6 m altitude, dust lifting was observed away from the takeoff or landing zone. Estimates of wind friction velocity at the time of onset of dust lifting were made using an existing sediment mobilization model. During landings at undisturbed locations, the onset of dust lifting was consistent with low‐speed saltation models, suggesting that sand may have been mobilized as well. Dust removal from a track downwind of the helicopter on flight 1 also suggests the initiation of a saltation cluster. Disturbed areas produced dust with lower modeled wind speeds, although source exhaustion was observed during a hover, followed by renewed dust lifting as the winds increased during landing. During traverses, dust lifting was observed from heights at which saltation was unlikely, suggesting the likelihood of break‐up of dust aggregates and entrainment of the resulting dust. Spatial heterogeneity was important: even within a single flight, dust lifting occurred in some areas but not in others. Recirculating dust clouds were able to form around the helicopter on at least two flights from a 5‐m cruising altitude, with an especially well‐developed one on flight 4. While the geometric conditions (such as height) for which dust lifting occurred were well‐constrained, a more accurate prediction of surface shear stress will require CFD modeling.

## Supporting information

Supporting Information S1

Movie S1

Movie S2

Movie S3

Movie S4

Movie S5

Movie S6

Movie S7

Movie S8

Movie S9

Movie S10

Movie S11

Movie S12

## Data Availability

All Perseverance data used in this study are publicly available via the Planetary Data System (PDS: Balaram, 2021; Bell & Maki, 2021; Maki, 2021; Rodriguez‐Manfredi & de la Torre Juarez, 2021). The derived data shown in Figures 6 and 8 are available in Lemmon (2022). The HiRISE mosaic shown in Figure 2 may be accessed via the Multi‐Mission Geographic Information System (Calef et al., 2020), and the original images are available via the PDS (McEwen, 2007).
