# Supplementary material for: Lifting and Transport of Martian Dust by the Ingenuity Helicopter Rotor Downwash as Observed by High‐Speed Imaging From the Perseverance Rover
Source: J Geophys Res Planets. 2022 Dec 20;127(12):e2022JE007605. doi: 10.1029/2022JE007605 (PMC10078181; doi:10.1029/2022JE007605)
Supplement: Supplementary file 1 — Supporting Information S1 [file JGRE-127-0-s012.pdf]

AGU PUBLICATIONS

*Journal of Geophysical Research*

Supporting Information for

# Lifting and transport of Martian dust by the Ingenuity helicopter rotor downwash as observed by high-speed imaging from the Perseverance rover

**M.T. Lemmon[1], R.D. Lorenz[2], J. Rabinovitch[3], C.E. Newman[4], N.R. Williams[5], R. Sullivan[6], M.P. Golombek[5], J.F. Bell III[7], J.N. Maki[5], A. Vicente-Retortillo[8]**

[1]Space Science Institute, Boulder, CO 80301, USA. [2]Applied Physics Laboratory, Laurel, MD 20723, USA. [3]Stevens Institute of Technology, Hoboken, NJ, 07030, USA. [4]Aeolis Research, Chandler, AZ, USA. [5]Jet Propulsion Laboratory, California Institute of Technology, Pasadena, CA, USA. [6]Cornell University, Ithaca, NY, USA. [7]Arizona State University, Tempe, AZ, USA. [8]Centro de Astrobiologia (INTA-CSIC), Madrid, Spain.

**Contents of this file**

Text S1 to S4

Figures S1 to S4

**Additional Supporting Information (Files uploaded separately)**

Captions for Movies SM01 to SM12

**Introduction**

Text S1 and Fig. S1 describe Earth-based testing of *Terry*, an *Ingenuity*-analog helicopter. Text S2 and Figs. S2-S3 describe dust (and other particulate) raising by terrestrial rotorcraft. Text S3 and Fig. S4 describe an alternate measurement of dust lifting on landing by Ingenuity that is more specific to the landing area and consistent with the reported results. Movies SM01-SM10 show dust-enhanced videos of *Ingenuity* flight segments.

**Text S1. Ingenuity downwash measurements.**

Tests were performed in September 2021 on a test unit of the Ingenuity helicopter, adapted for free flight in terrestrial conditions (hence its informal designation “Terry”). Its flight control system is completely different from Ingenuity (Terry is controlled with a joystick radio control rather than an autonomous autopilot), and the rotor speed is considerably lower than for Mars (due to the denser atmosphere on Earth). However, the rotor design is identical to Ingenuity, and thus, Reynolds number and Mach number effects aside, the downwash characteristics should be broadly representative of those on Mars, once scaled to the relevant rotor velocity and thrust. The tests were conducted principally to assess models for aeroacoustic emission, but the opportunity was taken to make some somewhat improvised downwash velocity measurements.

The Terry vehicle has a mass of 1.44 kg, and thus its weight on Earth is ~14N, compared with the 1.8 kg Ingenuity on Mars weighing 6.7 N. Despite the higher hover thrust requirement, the much denser terrestrial atmosphere means this thrust is generated with the rotors spinning at 400 rpm (+/- 2 rpm), compared with the 2400+ rpm for Ingenuity on Mars.

Four Kestrel 6500 handheld recording weather stations were mounted on short tripods such that their anemometers were about 25cm above the floor of the test facility (see Fig. S1). One was installed at a nominal distance of ~75cm from the rotor axis, just at the tip radius. The other stations were at progressively further distances of ~1.5, 2.5 and 3.5m away.

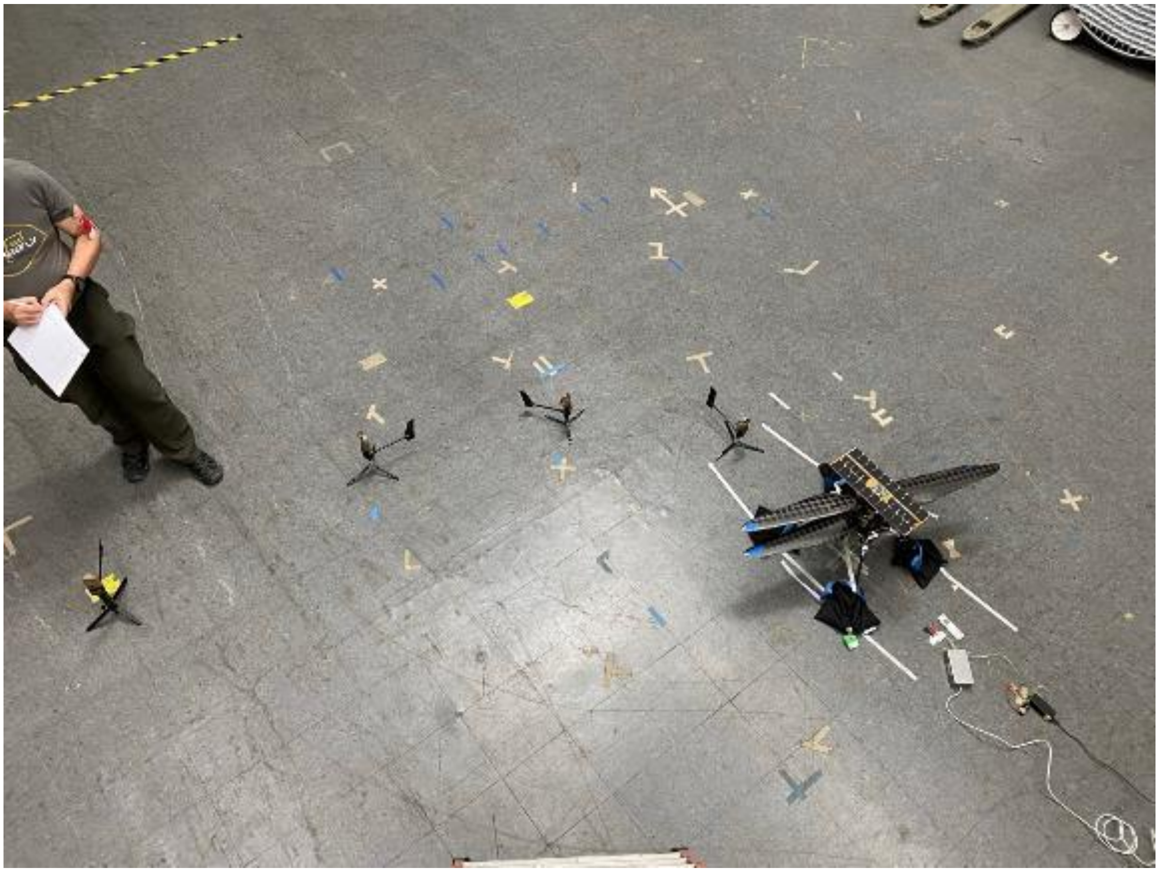

Figure S1 View from a stepladder of the anemometer array installation next to the Terry helicopter.

There was considerable (>30%) variation in the wind readings (recorded at 1/sec), in part due to the turbulent character of the downwash, presumably with a substantial tip vortex component, and in part due to the wandering of the hovering helicopter, whose horizontal position had excursions of several tens of centimeters due to the challenges of manual control. The altitude was maintained at ~1 and 2.5m for a couple of tens of seconds.

The outflow velocities measured by the anemometers were 1.5-2.8 m/s for the closest anemometer, and 0.7-1.4 m/s for the more distant ones, with no detectable gradient between them. The Rabinovitch et al. (2021) model was also adapted to Terry. For the closest anemometer, the predicted 1-m wind speed was 3.8 m $s^{-1}$. Thus, the model overpredicted wind speed by about a factor of 2 (1.4 to 2.5).

An Infiltec INFRA-20 microbarometer was used to record low frequency sound in related tests. Fluctuations at ~14 Hz (i.e., at the blade passage frequency) with a peak-to-peak variation of ~8 Pa were observed in hover. With the helicopter fixed to the floor, and the sensing pipe ~2m away (i.e., about 1.4m from the rotor tips), the fluctuations were ~4 Pa. These are presumably due to the cores of tip vortices being advected to the sensing location.

It is usefully instructive to observe that the 14 N weight of the helicopter must be borne by the rotor effects on the flowfield which are in turn expressed as a pressure distribution on the rotor blades. Taking the blade area as 2 x 1.2 x 0.1 = 0.24 $m^2$, it follows that the *average* pressure difference across the blades is ~ 14/0.24 ~ 60 Pa. In practice there is a strong spanwise variation of the pressure coefficient along the blade, and the value near the tip (where the pressure differential may roll out into a vortex) will be somewhat higher than the average value.

**Text S2. Dust raising by terrestrial rotorcraft**

We call attention in this appendix to terrestrial experience with dust-raising by rotorcraft downwash in Figs. S2-S3.

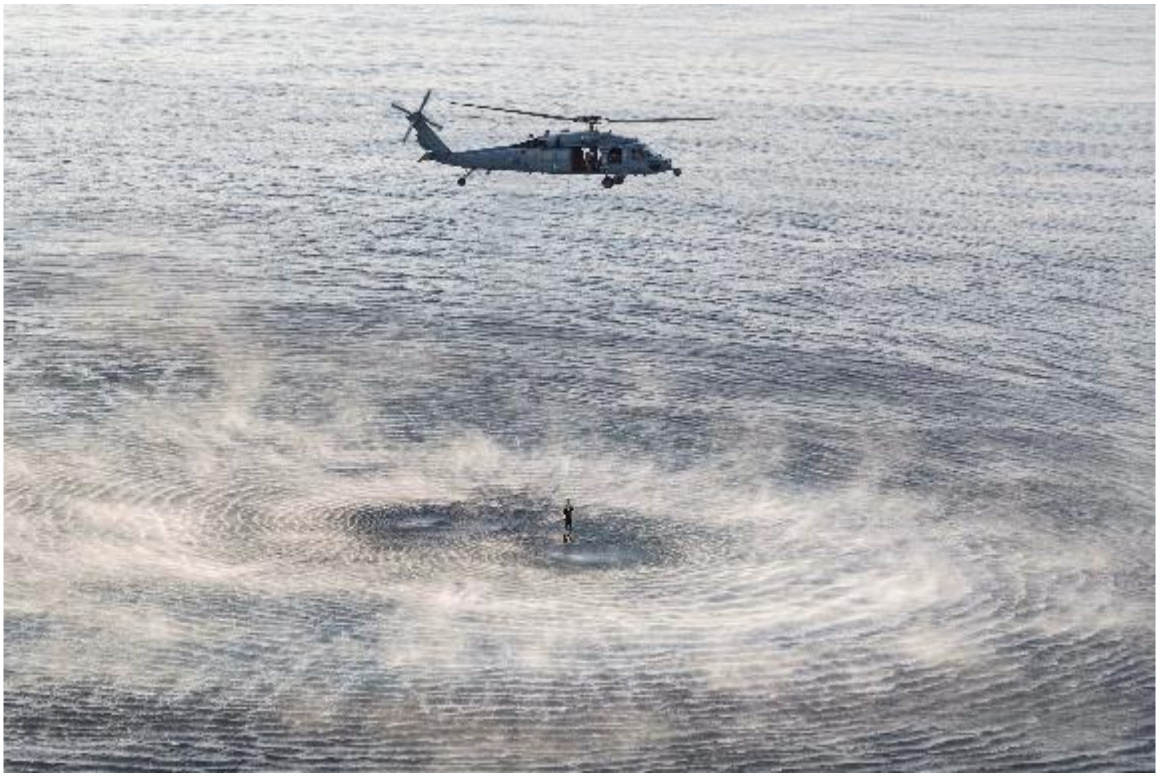

Figure S2.  A Navy MH-60S Seahawk helicopter during anti-mine countermeasures training in the Gulf of Aden, 2019. The ripples and spray provide a graphic indication of the shear stress imparted by the downwash a couple of diameters below and beyond the rotor disk. U.S. Department of Defense Photo 190410-N-HX510-0179Y by Navy Seaman Jarrod A. Schad.

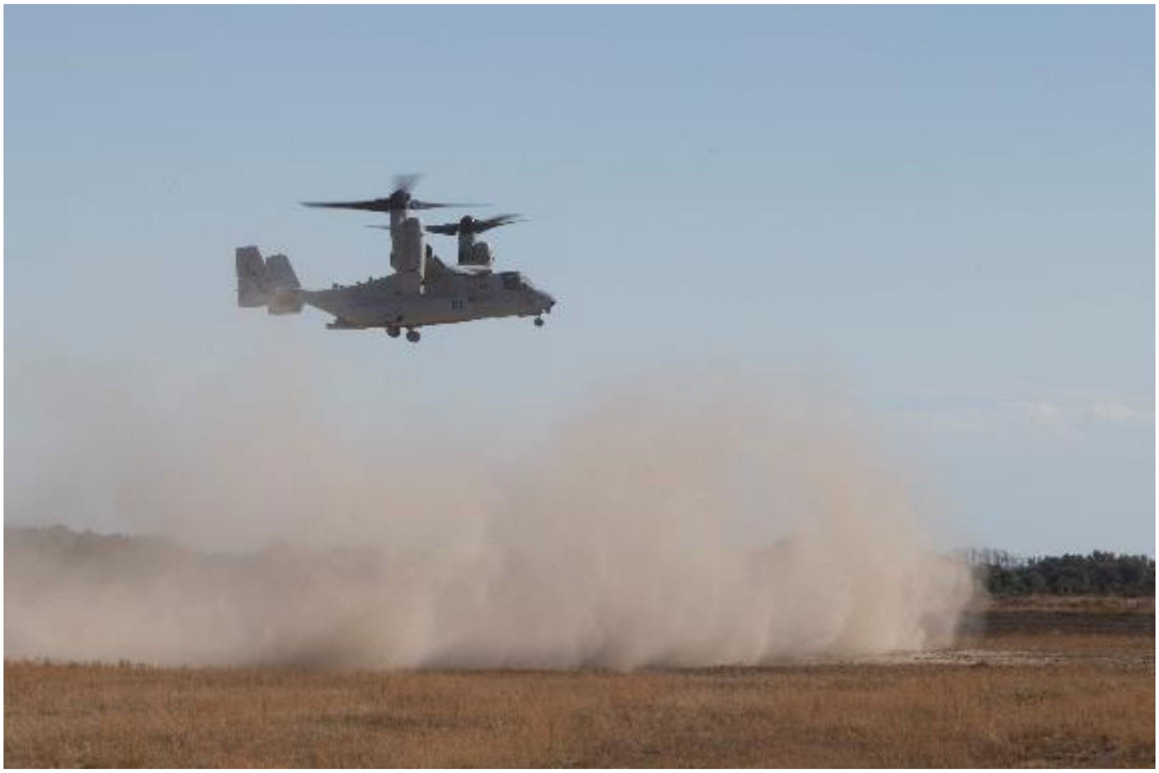

Figure S3. Although typical ‘brownout’ dust clouds generated by rotorcraft are only 2-3 rotor diameters across and form within one or two diameters of the ground, occasionally separated clouds can be seen. This one, with geometry very similar to that observed for Ingenuity at Jezero is a MV-22 Osprey tilt-rotor aircraft on exercises at Camp Lejeune.  US DoD Photo 171130-M-CO135-095 by U.S. Marine Corps Pfc. Ginnie Lee.

**Text S3. Lifted dust on landing detail.**

Rather than using the total lifted dust (i.e., Fig. 6), we measured the dust amount withing a 3-m by 1.5-m projected area around the landing site, shown in Fig. S4. This box avoided the noise added by many pixels away from landing and much of the dust raised prior to the landing sequence. In addition, due to the small size, it approximated a lifting rate averaged over some timescale of order 1 s as dust was rapidly advected out. Nonetheless, some dust remained, and we let the parameter k be fit, knowing that accumulating dust would modify that parameter.

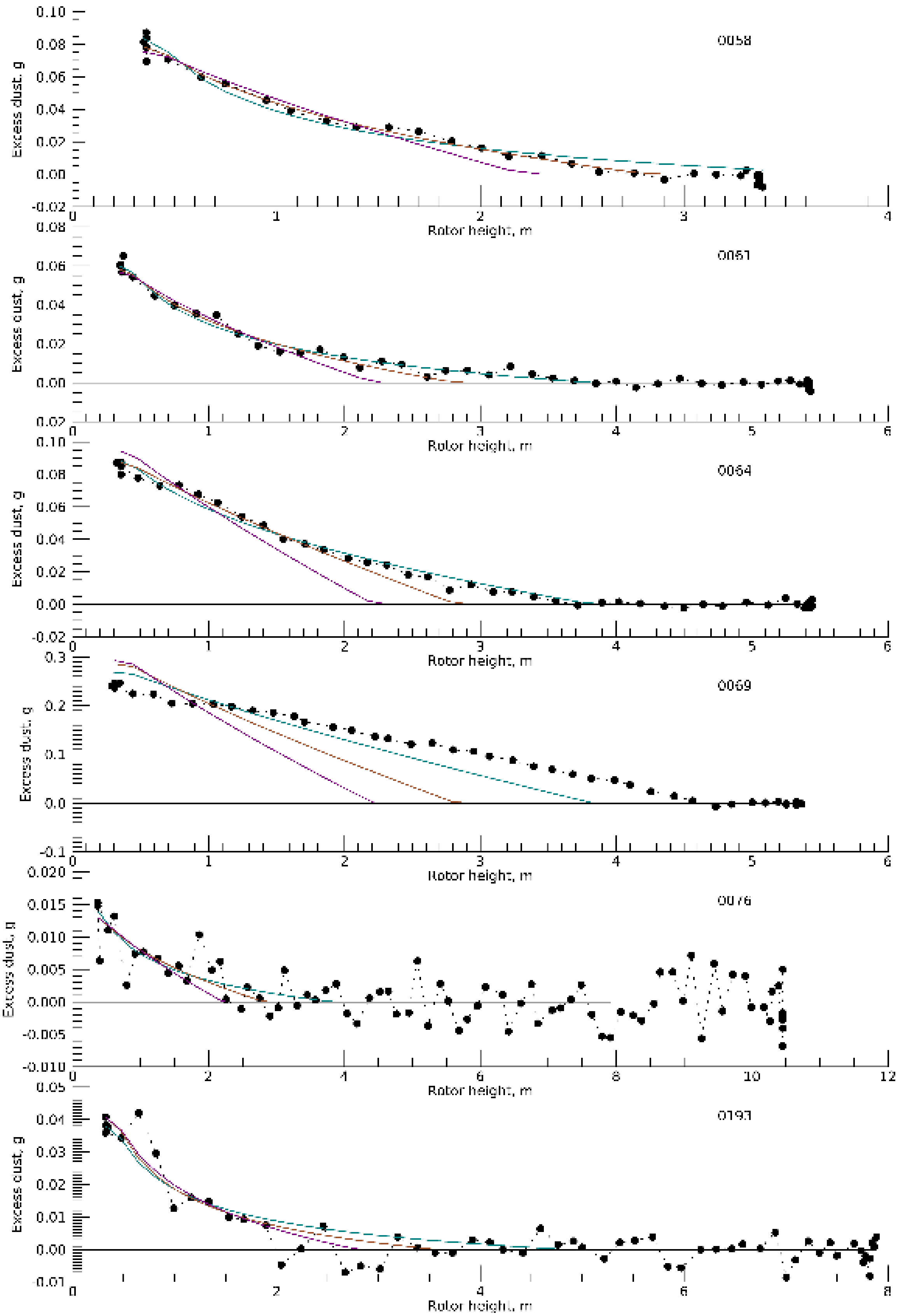
0058
0061
0064
0069
0076
0193
Excess dust, g
Rotor height, m

Figure S4. Lifted dust mass vs. altitude for landing within a 3x1.5 $m^2$ projected area. Colored curves are best-fit models for $u^*_t$ =0.8 (purple), 0.46 (brown), and 0.36 (teal) m/s.

**Text S4. Track formation.**

Figure S5 shows ratio images from before and after flights 1, 3, and 4 (sols 58, 64, and 69). Each before and after images was constructed as an average of available frames with no helicopter or near-filed dust cloud motion. Areas that got darker over the flight show up as dark in the image. Contours in the sky are due to the smooth gradient, with quantized brightness contours shifting with time. For flight 1 on sol 58 (left), the dust cloud shows up in the distance. Dust removal to the left of the helicopter (the slight displacement of the helicopter pre- and post-flight positions can be seen at bottom center) can be associated with the tilt of the helicopter in response to winds that lifted dust there. The dark streak that leads out of the frame to the right traces the path on which the take-off dust cloud departed but was never under the helicopter. Thus, the streak seems to indicate removal of bright surface dust downwind of the helicopter. On flights 3 and 4, the dark streak appears under the helicopters traverse. The dark area on the horizon on sol 69 was caused by a distant, bright dust devil in the ‘before’ image.

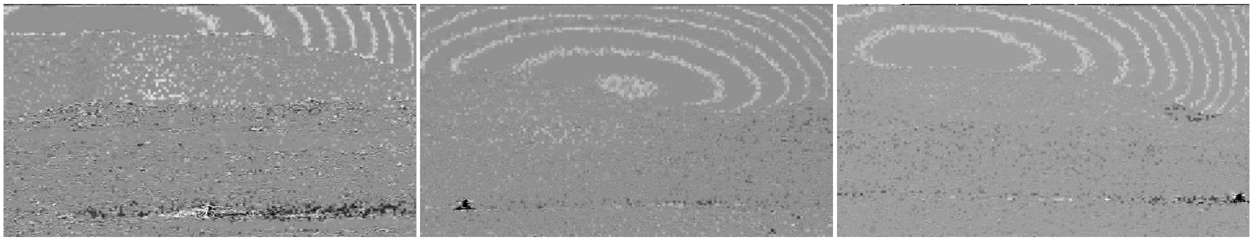

Figure S5. Before/after flight ratio images from sols (left to right) 58 (right eye), 64 (R), and 69 (L). Each image is constructed from the ratio of the green channel data with a 96-103% linear contrast stretch.

**Text S5Dust-enhanced movies.**

Movies showing the dust-enhanced frames from video of flight segments are included as separate files, SM1-SM12.

**Movie SM1.** Flight 1 on sol 58 with Mastcam-Z right eye video, enhanced as described and shown in text.

[01 https://gemelli.colorado.edu/~lemmon/flight_de_0058r.mp4 ]

**Movie SM2.** Flight 2 on sol 61 with Mastcam-Z right eye video, enhanced as described and shown in text.

[02 https://gemelli.colorado.edu/~lemmon/flight_de_0061r.mp4 ]

**Movie SM3.** Flight 3 on sol 64 with Mastcam-Z left eye take-off video, enhanced as described and shown in text.

[03 https://gemelli.colorado.edu/~lemmon/flight_de_0064l-to.mp4 ]

**Movie SM4.** Flight 3 on sol 64 with Mastcam-Z right eye take-off video, enhanced as described and shown in text.

[04 https://gemelli.colorado.edu/~lemmon/flight_de_0064r-to.mp4 ]

**Movie SM5.** Flight 3 on sol 64 with Mastcam-Z left eye landing video, enhanced as described and shown in text.

[05 https://gemelli.colorado.edu/~lemmon/flight_de_0064l-td.mp4 ]

**Movie SM6.** Flight 3 on sol 64 with Mastcam-Z right eye landing video, enhanced as described and shown in text.

[06 https://gemelli.colorado.edu/~lemmon/flight_de_0064r-td.mp4 ]

**Movie SM7.** Flight 4 on sol 69 with Mastcam-Z left eye take-off video, enhanced as described and shown in text.

[07 https://gemelli.colorado.edu/~lemmon/flight_de_0069l-to.mp4 ]

**Movie SM8.** Flight 4 on sol 69 with Mastcam-Z right eye take-off video, enhanced as described and shown in text.

[08 https://gemelli.colorado.edu/~lemmon/flight_de_0069r-to.mp4 ]

**Movie SM9.** Flight 4 on sol 69 with Mastcam-Z left eye landing video, enhanced as described and shown in text.

[09 https://gemelli.colorado.edu/~lemmon/flight_de_0069l-td.mp4 ]

**Movie SM10.** Flight 4 on sol 69 with Mastcam-Z right eye landing video, enhanced as described and shown in text.

[10 https://gemelli.colorado.edu/~lemmon/flight_de_0069r-td.mp4 ]

**Movie SM11.** Flight 5 on sol 76 with Mastcam-Z right eye landing video, enhanced as described and shown in text.

[11 https://gemelli.colorado.edu/~lemmon/flight_de_0076r-td.mp4 ]

**Movie SM12.** Flight 13 on sol 193 with Mastcam-Z right eye video, enhanced as described and shown in text.

[12 https://gemelli.colorado.edu/~lemmon/flight_de_0193r.mp4 ]
